# Post-stroke dysphagia is associated with abnormal gamma power and phase synchronisation during swallowing

**DOI:** 10.3389/fnhum.2026.1716564

**Published:** 2026-02-13

**Authors:** Lingyan Wang, Shengqiao Wang, Heliang Yang, Qiwei Li, Jiong Feng, Ying Zhao, Hong Hong, Qingchuan Gu, Xianwei Che, Aiqun Shi, Jiasheng Wang

**Affiliations:** 1 Department of Rehabilitation Medicine, Jinhua TCM Hospital Affiliated to Zhejiang Chinese Medical University, Jinhua, China; 2 Centre for Cognition and Brain Disorders, The Affiliated Hospital of Hangzhou Normal University, Hangzhou, China; 3 Department of Encephalopathy, Jinhua TCM Hospital Affiliated to Zhejiang Chinese Medical University, Jinhua, China; 4 Department of Neurology, The Affiliated Hospital of Hangzhou Normal University, Hangzhou, China

**Keywords:** dysphagia, EEG, neuromodulation, oscillation, stroke, swallowing

## Abstract

Post-stroke dysphagia (PSD) has a series of complications that are associated with poor recovery and even mortality. Uncovering abnormal neural activities during swallowing bears critical implications for optimising treatment strategies for PSD patients. The current study was designed to identify the abnormal neural representations of swallowing in PSD. PSD individuals, stroke individuals without dysphagia, and healthy controls underwent a swallowing task while ongoing EEG was recorded. Compared to healthy controls, PSD patients demonstrated predominant gamma-band hypoactivity and hypoconnectivity during swallowing. We also identified an excessive gamma-band hyperconnectivity that was related to more severe dysphagia, possibly reflect inefficient neural effort or muscle recruitment during swallowing. As a secondary outcome, PSD patients demonstrated lower theta-band connectivity compared to those without dysphagia. These novel findings may help inform future research on potential neuromodulation approaches for PSD. However, this is considered as a pilot study, and these findings should be explained in the context of a small sample size.

## 1 Introduction

Post-stroke dysphagia (PSD) is a debilitating condition affecting 30–65% of stroke survivors (Martino et al., 2005). There are a series of complications of PSD, such as aspiration pneumonia, malnutrition, and dehydration. Therefore, dysphagia was found to be associated with prolonged hospital stays, poor recovery, and increased mortality (Altman et al., 2010; González-Fernández et al., 2013).

In terms of treatment for PSD, postural adjustments (e.g., chin-tuck) and bolus consistency modification are widely used in clinical practices (Speyer et al., 2010; Warnecke et al., 2021). In addition, non-invasive neuromodulation technologies have been proposed as promising treatment strategies for dysphagia, such as peripheral electrical stimulation (PES), transcutaneous neuromuscular stimulation (TNS), repetitive transcranial magnetic stimulation (rTMS), and transcranial direct current stimulation (tDCS; Ludlow et al., 2007; Suntrup et al., 2015; Vasant et al., 2014; Verin and Leroi, 2009). Although some of these techniques demonstrated a positive effect on dysphagia, the treatment efficacy remains suboptimal (Cheng et al., 2022; Sasegbon et al., 2020).

Understanding the oscillatory mechanisms of PSD bears critical implications to optimise the treatment efficacy for neuromodulation. For instance, transcranial alternating current stimulation (tACS) and rTMS could be personalised to entrain neural oscillations with the purpose to increase treatment efficacy. Unfortunately, the abnormal neural representations in PSD remain largely unknown. Using electroencephalogram (EEG), one study demonstrated gamma oscillation to support voluntary swallowing in epileptic participants (Hashimoto et al., 2021a). In another study, gamma power was found to be coupled with alpha phase during swallowing (Hashimoto et al., 2021b). In healthy controls, gamma oscillation was also found to discriminate different head positions that require varying effort during swallowing (Jestrović et al., 2015). These novel findings indicate gamma oscillation as a potential oscillatory mechanism of swallowing. Yet, the role of gamma oscillation in PSD patients remains to be established. The current study thus represents the inaugural EEG study of swallowing in post-stroke dysphagia to underscore its significance. Beyond gamma activity, a few other studies have suggested that alpha and beta oscillations are also important for swallowing control (Teismann et al., 2009; Cuellar et al., 2016; Koganemaru et al., 2021; Imaz-Higuera et al., 2025).

The objective of the current study was to identify abnormal neural representations of swallowing in PSD, focusing on gamma-band power and connectivity. Participants underwent a swallowing task while ongoing EEG was recorded. It is hypothesised that PSD patients would demonstrate lower gamma power during swallowing. Findings from this investigation would identify neural representations that provide insights to optimise brain stimulation technologies for PSD treatment.

## 2 Methods

### 2.1 Participants

This pilot study included 10 patients (8 males and 2 females, mean age 60.6 ± 11.2 years) diagnosed with post-stroke dysphagia. The inclusion criteria were: (1) meeting the diagnostic criteria for stroke as defined in the ‘Diagnostic Key Points for Cerebrovascular Diseases (China National Conference on Cerebrovascular D, 1995) issued by the Chinese Society of Neurology, and confirmed by cranial CT or MRI; (2) with clear consciousness and able to cooperate with swallowing; (3) first-time stroke or with a history of stroke but without previous dysphagia, (4) with a disease course within 3 months; (5) with stable vital signs and no severe complications in organs such as the heart and lungs. The exclusion criteria were: (1) having a history of dysphagia; (2) significant brain edema confirmed by CT or MRI; (3) having recently taken medications that affect swallowing function (such as diuretics, muscle relaxants); (4) with local pharyngeal diseases or a history of pharyngeal surgery. We also recruited 10 stroke patients without dysphagia (9 males, 1 female, mean age 58.6 ± 10.58 years). For these patients, the absence of swallowing dysfunction was confirmed by the Video fluoroscopic Swallowing Study (VFSS) at admission, with a Penetration-Aspiration Scale (PAS) grade of 1. Otherwise, the same inclusion and exclusion criteria were applied to them. Healthy controls were also recruited to match the patients in age (55.40 ± 9.98 years, *p* = 0.560) and gender [7 males and 3 females, χ^2^(2) = 1.250 *p* = 0.535] for both patient groups. This study was approved by the Ethics Committee of Jinhua Hospital of Traditional Chinese Medicine, Zhejiang Province (20190606003). All participants signed an informed consent before the commencement of this study.

### 2.2 Study design

This was an observational study. Clinical assessments included the Swallowing Severity Assessment (SSA; Perry, 2001), the Penetration-Aspiration Scale (PAS; Rosenbek et al., 1996), the National Institutes of Health Stroke Scale (NIHSS; Brott et al., 1989), the Mini-Mental State Examination (MMSE; Folstein et al., 1975), and the Dysphagia Outcome and Severity Scale (DOSS; O'Neil et al., 1999). EEG data were also collected during a swallowing task.

### 2.3 Swallowing task and EEG data collection

Before EEG acquisition, each subject was asked to maintain a sitting position and practice the swallowing task. Subsequently, water was mixed with Otsuka Swallow, a medical thickening agent (Guangzhou Audite Biotechnology Co., Ltd. GB/T29602) to form a grade 2 thick liquid. Two millilitres of the thickened milk was administered onto the middle of the subject’s tongue while an event-related marker was concurrently sent to the EEG system. This administration marker was used only to document task timing and was not used for EEG epoching because the delay before swallowing varies across individuals. Another marker was sent when the subject finished swallowing. This marker was used to confirm the end of the behavioural act but was not used as a boundary for EEG segmentation. To accurately determine the true swallowing boundaries, we additionally identified swallow onset and offset using two complementary physiological indicators. Swallow onset was defined as the earliest time point at which either laryngeal elevation was visually observed by an experienced clinician, or the suprahyoid surface EMG (sEMG) signal showed a clear deviation from baseline. This onset event served as the starting point (time = 0) for EEG epoching. Swallow offset was defined as the completion of laryngeal elevation, indicated by the return of the larynx to its resting position and confirmed by the corresponding return of the suprahyoid sEMG activity to baseline. The offset marker helped verify that the swallowing movement fell within the analysis window but was not used to define the EEG epoch. This procedure was repeated 50 times to get enough trials. The same task was performed by healthy controls. During the swallowing task, there were minimal pauses, and any brief interruption, was considered as the rest period. This pause was typically only a few seconds long. In case of coughing, the swallowing task was paused until the subject recovered, and the task resumed once the subject was ready.

The EEG signals were collected using a Nihon Kohden system (EEG-1200C) following the international 10–20 channel layout, with reference to electrodes A1 and A2. The device had 32 recording channels, with a sampling rate of 500 Hz. The impedances were kept below 5 kΩ throughout the recordings. Only scalp EEG electrodes were included for subsequent analysis. Physiological monitoring channels (e.g., EMG, ECG, CO₂, pulse, and SpO₂ signals) and redundant auxiliary channels provided by the acquisition system were excluded because they did not record cortical electrical activity. After removing these non-EEG channels, a total of 19 electrodes were used for recording (Fp1, Fp2, F7, F8, F3, Fz, F4, T3, C3, Cz, C4, T4, P3, Pz, P4, T5, T6, O1, O2).

### 2.4 EEG preprocessing and analysis

EEG data were pre-processed offline using custom-written scripts that implement functions from EEGLAB (version 15.0; Delorme and Makeig, 2004) running under Matlab R2022b (The MathWorks, Inc.). Data from malfunctioning channels were visually inspected and removed. Data were bandpass filtered between 0.1 and 100 Hz and bandstop filtered between 48 and 52 Hz using zero-phase Butterworth filters (Widmann et al., 2015). Continuous data were segmented based on the swallow onset of each trial, which was identified using the earliest laryngeal elevation or the first deviation of the suprahyoid sEMG signal from baseline. EEG epochs were extracted from −1 to +6 s relative to this swallow onset. Segmented data were re-referenced to the average reference, and the fast independent component analysis algorithm (FastICA) was used to remove stereotyped artefacts, e.g., eye blinks, lateral eye movements, muscle, and line noise (Korhonen et al., 2011). Stereotyped artefacts were identified by visual inspection of the temporal and spatial representation of the independent components. Missing channels were then interpolated, and epochs were inspected again to remove any anomalous activity in the signal. All swallow trials were pooled for analysis except for the rejected trials in pre-processing.

Time-frequency analyses were performed using hanning tapered “mtmconvol” method in the FieldTrip toolbox (Oostenveld et al., 2011). Power was calculated in the range of 1–100 Hz in the time window of −1 to 6 s and baseline corrected (−1 s) for each trial before averaging trials in each condition for each subject.

EEG connectivity was calculated by computing the debiased weighted phase lag index (wPLI) based on the frequency representations obtained above. The wPLI is a measure of the phase coherence of two signals, based on the imaginary part of the cross-spectrum (Che et al., 2019; Vinck et al., 2011). WPLI is suggested to be able to reduce sensitivity to additional, uncorrelated noise sources, such as volume conduction. It is also able to increase statistical power to detect changes in the phase-synchronisation (Vinck et al., 2011). For each frequency steps, wPLI was computed for each electrode pair. Connectivity values were then averaged to the frequency and time frames of interest based on results from the power analysis (i.e., gamma). Other frequency bands were further examined for exploratory purposes. To calculate wPLI, hanning tapered “mtmconvol” method was initially used for time-frequency analyses, in which the output was specified to ‘fourier’ to generate complex fourier-spectra and then baseline corrected (−1 s).

### 2.5 Statistical analysis

For time-frequency data, non-parametric cluster-based permutation statistics were performed at a global level. This method is able to control multiple comparisons across space and time (Maris and Oostenveld, 2007). The cluster-based permutation tests were applied to the time window of interest (0–6 s) in all channels. Subsequent pairwise t-tests were then conducted for comparison. An observed test statistics value was considered in the cluster permutation if it was below the threshold of 0.05 in at least 2 of the neighbouring channels (Oostenveld et al., 2011). We performed 5,000 iterations of trial randomisation for generating the permutation distribution, controlling for multiple comparisons across space (*p* < 0.05). Statistical analysis was initially performed in gamma (31–100 Hz) band based on *a prior* hypothesis. Exploratory analyses were also conducted in other frequency bands (i.e., delta: 1–3 Hz, theta: 4–7 Hz, alpha: 8–12 Hz, beta: 13–30 Hz).

**Figure 1:**
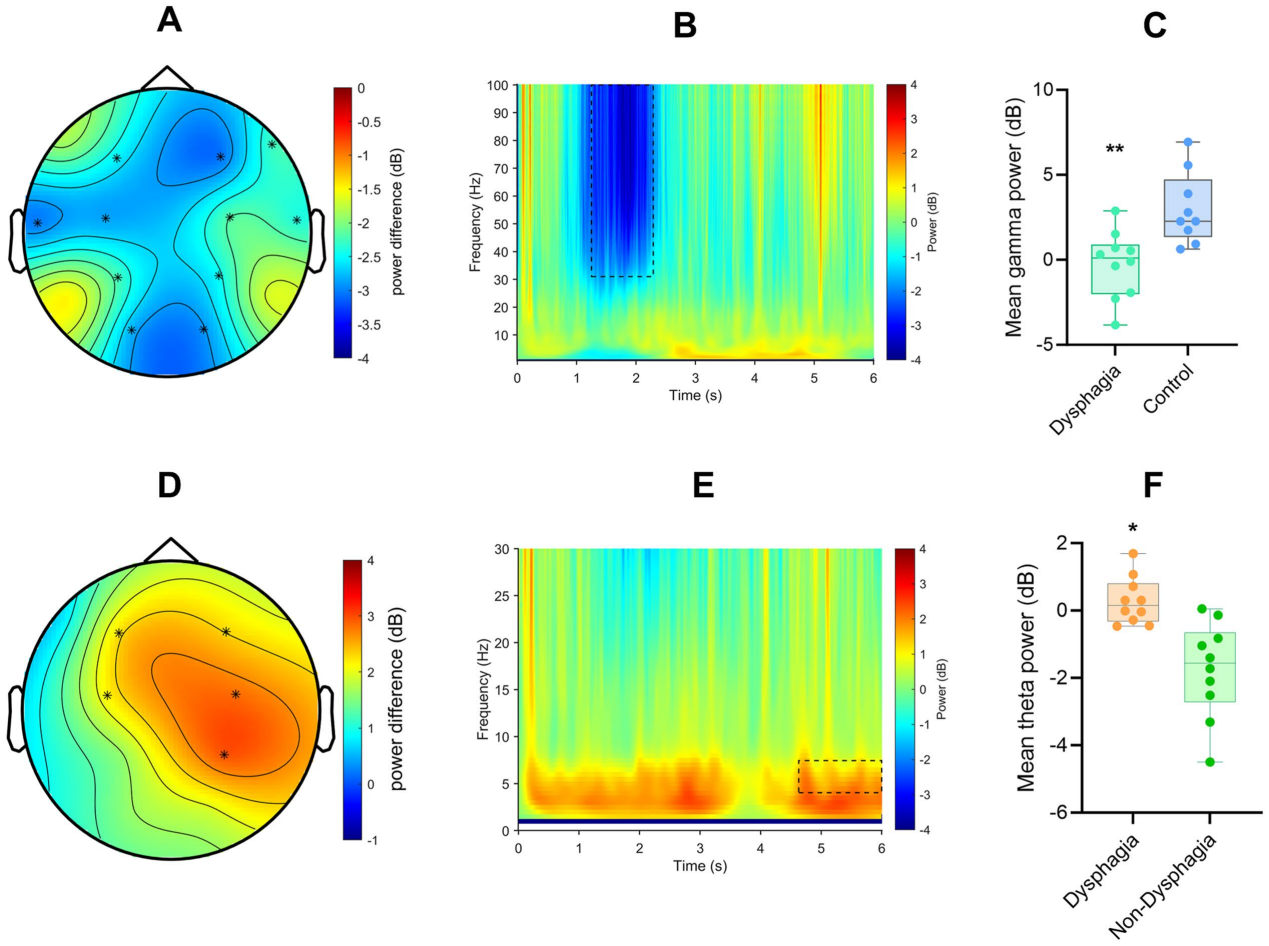
**(A)** Topographical distribution of gamma power differences (patients vs. healthy controls) in the frontal, central, and parietal regions. **(B)** Time-frequency representation of gamma power difference observed between 1.25–2.29 s (patients vs. healthy controls, averaged from all 19 channels). **(C)** Averaged values of the gamma power cluster extracted across significant time and channels (patients vs. healthy controls). **indicates *p* < 0.01. **(D)** Topographical distribution of theta power differences (post-stroke PSD patients vs. post-stroke patients without dysphagia) in the frontal, central, and parietal regions. **(E)** Time-frequency representation showing theta power difference observed between 4.63 and 6 s (PSD patients vs. post-stroke patients without dysphagia, averaged from all 19 channels). **(F)** Averaged power values of theta band extracted from the significant time windows and channels (PSD patients vs. post-stroke patients without dysphagia). * indicates *p* < 0.05. ** indicates *p* < 0.01.

To visualise individual subject values (Figures 1C,F), mean power was extracted only from the significant spatiotemporal cluster identified by the cluster-based permutation analysis, ensuring that the plotted data corresponded directly to the statistical test rather than to whole-scalp averages.

Statistical analyses of EEG connectivity were performed in SPSS (v.25.0 Chicago, Illinois). Based on the results of time-frequency analysis, comparisons were made between groups that showed significant oscillatory difference as *a priori* strategy. Group comparisons were performed for gamma (1.25–2.29 s) and theta-band (4.63–6.00s) wPLI across all 171 unique electrode pairs (19 × 18/2). Given the pilot nature of the study and the modest sample size, these edge-wise connectivity comparisons were treated as exploratory and are reported at a threshold of *p* < 0.05.

We further performed correlation analyses between EEG connectivity indices and swallowing performance. Because DOSS scores were non-normally distributed based on the Shapiro–Wilk test, Spearman’s rank correlation coefficient was used. Correlations with DOSS were computed for 10 gamma-band connections that showed nominal group differences in the analyses. To control for multiple correlations, a Bonferroni-corrected significance threshold of *α* = 0.005 (0.05/10) was applied.

## 3 Results

### 3.1 Clinical information

Table 1 presents an overview of the clinical characteristics of the enrolled patients. The majority of the patients were male (8/10 males in the dysphagia group, and 9/10 in the non-dysphagia group), with a mean age of 60.60 ± 11.20 years for the dysphagia group and 58.60 ± 10.60 years for the non-dysphagia group. The mean disease duration was 1.75 ± 0.82 months for the dysphagia group and 2.47 ± 0.92 months for the non-dysphagia group. More than half of the patients (6/10 in the dysphagia group and 6/10 in the non-dysphagia group) had lesions in subcortical regions.

**Table 1:** Demographic and clinical information of patients.

ID	Gender	Age	Lesion	Disease duration (month)	SSA	PAS	MMSE	NIHSS	DOSS	Group
1	Male	45	Right thalamus and basal ganglia	0.70	26	8	18	11	4	Dysphagia
2	Male	44	Left frontal and temporal lobes	1.10	31	4	8	12	6	
3	Male	55	Left thalamus	1.77	25	4	20	12	5	
4	Male	60	Right parietal-occipital lobe	1.30	23	4	19	17	5	
5	Female	74	Right fronto-parieto-temporal lobes	2.53	22	5	20	15	5	
6	Male	51	Right basal ganglia	1.70	19	3	26	10	4	
7	Female	71	Right frontal lobe	2.53	31	6	14	13	5	
8	Male	71	Left basal ganglia	1.17	21	4	18	11	4	
9	Male	68	Periventricular region	3.00	22	4	17	17	4	
10	Male	67	Left basal ganglia	1.30	20	3	15	10	4	
1	Male	65	Right thalamus and basal ganglia	3.00	26	1	20	11	7	Non-dysphagia
2	Male	35	Left frontal and temporal lobes	2.30	27	1	10	12	7	
3	Male	55	Left thalamus	3.00	25	1	21	12	7	
4	Male	61	Right parietal-occipital lobe	1.70	24	1	19	17	7	
5	Female	61	Right fronto-parieto-temporal lobes	2.30	25	1	22	15	7	
6	Male	60	Right basal ganglia	2.83	26	1	19	10	7	
7	Male	47	Right frontal lobe	0.60	24	1	20	13	7	
8	Male	73	Left basal ganglia	3.00	24	1	18	11	7	
9	Male	71	Right periventricular region	2.47	25	1	25	17	7	
10	Female	58	Left basal ganglia	2.36	24	1	16	10	7	

SSA, Swallowing Severity Assessment; PAS, Penetration-Aspiration Scale; NIHSS, National Institutes of Health Stroke Scale; MMSE, Mini-Mental State Examination; DOSS, Dysphagia Outcome and Severity Scale.

Independent-samples t-test revealed a significant difference between the dysphagia and non-dysphagia groups in PAS (t(18) = 7.33, *p* < 0.001, Cohen’s d = 2.32) and DOSS (t(18) = −7.80, *p* < 0.001, Cohen’s d = −2.47). In contrast, no significant difference was found in SSA (t(18) = −0.72, *p* = 0.486), MMSE (t(18) = 0.77, *p* = 0.449) scores. For NIHSS scores, which violated the assumption of normality, a Mann–Whitney U test was performed and indicated no significant difference between groups (U = 54.50, *p* = 0.739). Meanwhile, the healthy controls were matched with the two patient groups in terms of gender and age.

### 3.2 Time-frequency analysis

Cluster-based permutation tests revealed a gamma inhibition in PSD patients compared to healthy controls during the swallowing task (*t* = −2.11, *p* = 0.003, time range = 1.25–2.29 s, Cohen’s d = −1.474). This cluster was widely distributed over the frontal, central, and parietal regions. Additionally, there was group differences in theta oscillations. PSD patients had higher theta power than those without dysphagia (*t* = 4.33, *p* = 0.024, time range = 4.63–6.00s, Cohen’s d = 0.763) during the swallowing task (Figure 1). Although some non-significant contrasts showed visually apparent fluctuations (e.g., delta activity), these effects did not form spatially contiguous clusters across neighbouring electrodes.

To ensure comparability across groups, we performed full pairwise comparisons among the three groups (PSD patients, stroke patients without dysphagia, and healthy controls) across all canonical frequency bands (delta, theta, alpha, beta, and gamma) using the same cluster-based permutation framework. Only two contrasts yielded significant clusters: gamma-band power differences between PSD patients and healthy controls, and theta-band power differences between PSD patients and stroke patients without dysphagia. Importantly, no significant oscillatory differences were observed between stroke patients without dysphagia and healthy controls across all frequency bands (all cluster-level *p* > 0.05), underscoring the specificity of the identified abnormalities to the dysphagic condition.

### 3.3 EEG connectivity and correlations

**Figure 2:**
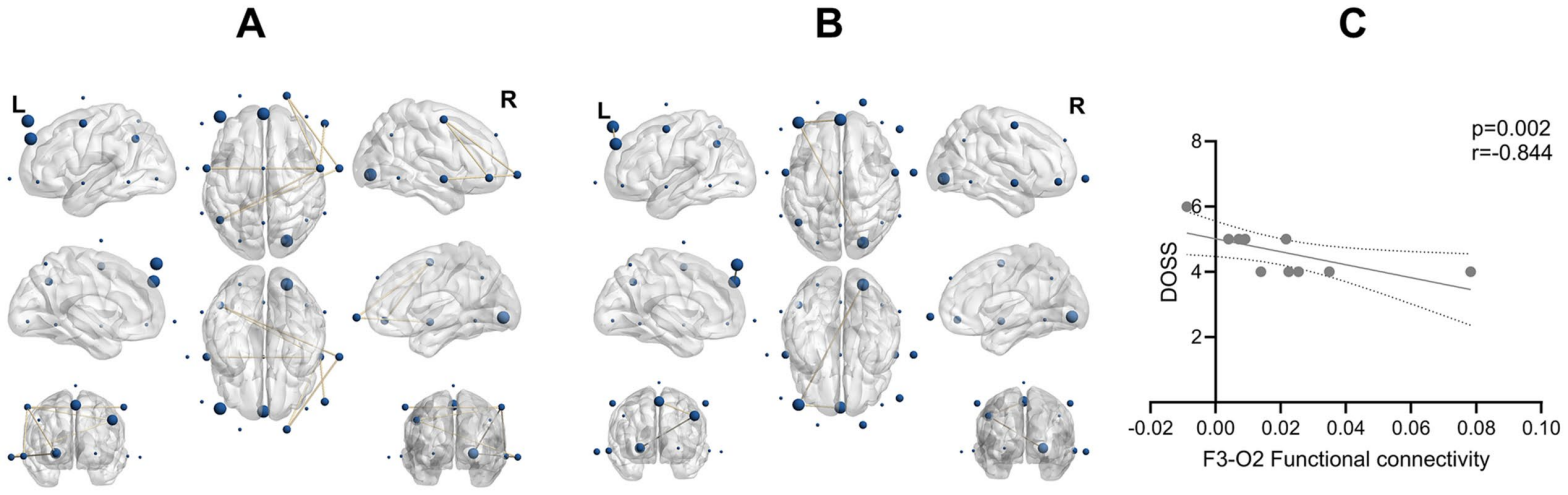
**(A)** Patients had lower gamma band connectivity during the swallowing task. EEG connectivity analysis revealed significantly lower gamma band connectivity in patients compared to healthy controls (*p* = 0.012–0.042). The figure shows only connections with statistically significant group differences. **(B)** EEG connectivity analysis revealed two gamma band connections (F3–O2, *p* = 0.016; F3–Fz, *p* = 0.024) with significantly higher connectivity in patients compared to healthy controls. **(C)** A significant negative correlation was found between the F3–O2 connectivity and DOSS scores (*r* = −0.844, *p* = 0.002, 95% CI = [−0.962, −0.458]), indicating that increased connectivity in this circuit was associated with poorer swallowing function.

In line with event-related oscillations, PSD patients demonstrated lower gamma band connectivity compared to healthy controls (*t* = 2.285–2.827, *p* = 0.012–0.042, Cohen’s d = [−1.307, −1.050]), between the frontocentral (Fp2, F8, C3, C4) and centroparietal regions (C3, C4, P3, T4). In addition, two gamma connections, F3–O2 (*t* = −2.671, *p* = 0.016) and F3–Fz (*t* = −2.486, *p* = 0.024), demonstrated higher connectivity in PSD patients than healthy controls. Furthermore, a significant negative correlation was observed between the F3–O2 and connectivity and DOSS scores (*r* = −0.844, *p* = 0.002), indicating that stronger connectivity was associated with poorer swallowing performance (Figure 2).

In terms of theta connectivity, PSD patients demonstrated lower theta-band connectivity compared to those without dysphagia (*t* = [2.254, 3.133], *p* = 0.006–0.037, Cohen’s d = [−1.401,-1.008]), mainly between the frontotemporal regions (F7, F8, Fp1, F4, T3, T4, T6) and the parietal region (P3). Additionally, F3–O1 connectivity was higher in PSD patients compared to those without dysphagia (*t* = −2.814,*p* = 0.011, Cohen’s d = 1.259). Notably, the mean gamma power—although significantly different between PSD patients and healthy controls—was not associated with DOSS within PSD patients (Spearman *ρ* = 0.107, *p* = 0.768, *n* = 10). No other significant group differences or correlations were observed.

### 3.4 Supplementary analysis

**Figure 3:**
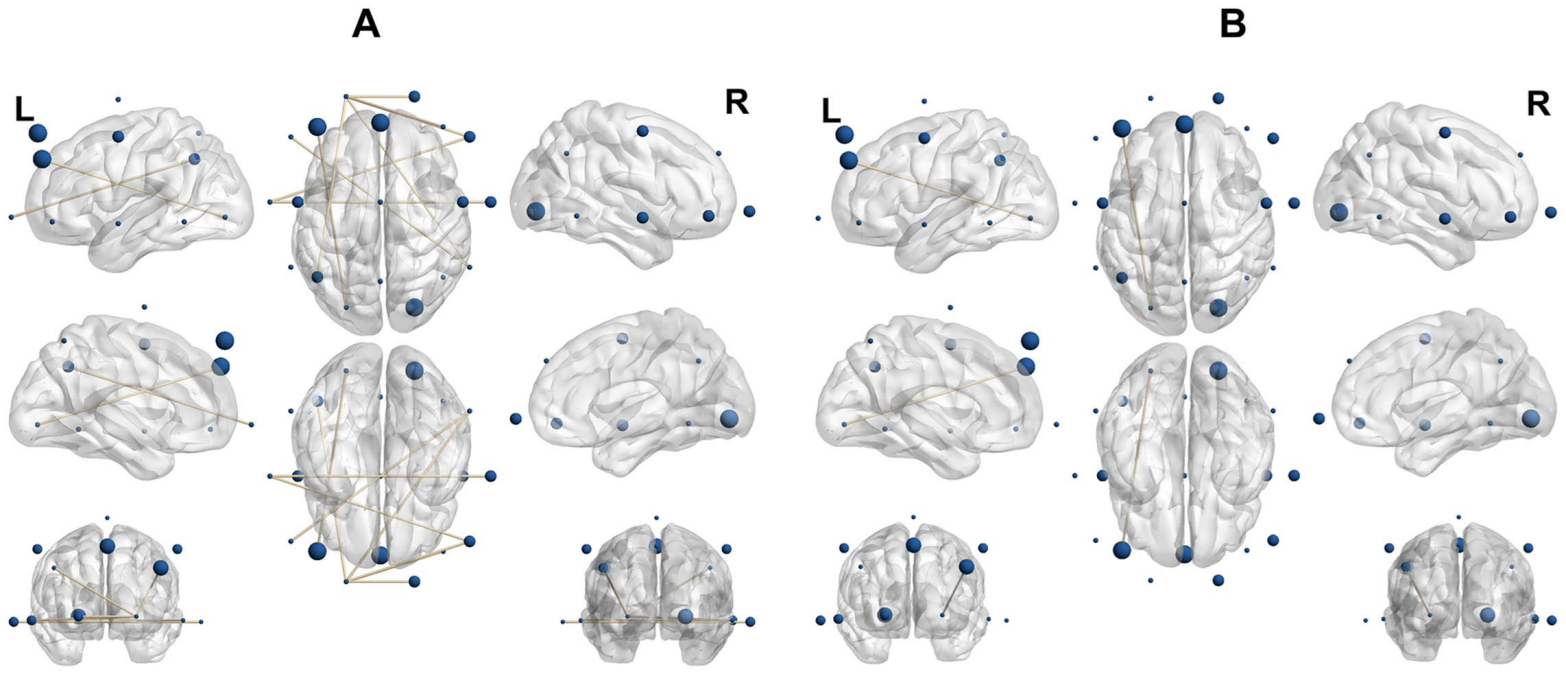
PSD patients show weaker theta band connectivity than those without dysphagia. **(A)** EEG connectivity analysis revealed significantly lower theta band connectivity in patients with dysphagia compared to those without dysphagia (*p* = 0.006–0.037). The figure shows only connections with statistically significant group differences. **(B)** F3-O1 connectivity is stronger in patients with dysphagia compared to those without dysphagia. EEG connectivity analysis revealed significantly higher F3-O1 connectivity in PSD patients compared to those without dysphagia (*p* = 0.011).

Additional analyses were performed to examine whether age and sex influenced the observed oscillatory abnormalities. For the gamma-band differences between PSD patients and healthy controls, a linear model was conducted with gamma-band mean power as the dependent variable, group as the fixed factor, and age and sex as covariates. Neither sex (F(1,15) = 0.003, *p* = 0.959) nor age (F(1,15) = 0.209, *p* = 0.654) showed significant effects on gamma power. Importantly, the group difference remained significant after adjustment (F(1,15) = 7.019, *p* = 0.018, partial η^2^ = 0.319). These findings indicate that the gamma-band hypoactivity observed in PSD patients cannot be attributed to demographic imbalance (Figure 3).

## 4 Discussion

Our data indicated that PSD patients demonstrated hypoactivity in gamma oscillation during swallowing. EEG studies have identified gamma oscillations as crucial for supporting voluntary swallowing, with evidence from studies in epileptic participants (Hashimoto et al., 2021a). Furthermore, phase-amplitude coupling analysis has revealed that gamma power is modulated by the phase of alpha oscillations during swallowing, suggesting a coordinated cross-frequency interaction (Hashimoto et al., 2021b). Our data extend this evidence to PSD patients, identifying a widely distributed gamma hypoactivity during swallowing compared to healthy controls. Gamma-band activity has been consistently linked to large-scale cortical communication and sensory-motor integration, thereby supporting its role as a critical neural substrate for the coordination of swallowing-related processes (Fries, 2015).

We also provided novel evidence that PSD patients had hypoconnectivity in the gamma range between distributed regions. This pattern of network hypoconnectivity was centred in the central cortices, with long-range connections with the frontal and parietal regions. This frontocentral and centroparietal networks advance our understanding of gamma oscillation in dysphagia, as previous studies either recorded intracranial EEG in specific locations or extracted small-world EEG properties with limited spatial information (Hashimoto et al., 2021a; Hashimoto et al., 2021b; Jestrović et al., 2015). Beyond EEG recordings, this network hypoconnectivity is consistent with findings from magnetic resonance imaging (MRI), whereby swallowing generally activates brain regions in the sensorimotor and parietal cortex (Cheng et al., 2022; Malandraki et al., 2009). Phase-based connectivity measures such as the weighted phase-lag index provide robust estimates of functional communication between cortical regions while minimising volume conduction effects, and are widely applied in motor and cognitive network research (Vinck et al., 2011; Fries, 2015) Time-frequency EEG analysis separates power and phase information across frequencies to provide closer interpretations of neural dynamics, capturing both transient event-related and sustained oscillatory processes that may be overlooked by traditional time-domain or Fourier-based approaches (Morales and Bowers, 2022). Furthermore, phase-lag index (PLI) and its variants such as the weighted phase-lag index (wPLI) are widely used to quantify phase synchronisation while reducing biases from volume conduction and common source effects, as these indices evaluate non-zero phase lags between signals, making them robust measures for true functional connectivity estimation in EEG (Stam et al., 2007). These connectivity metrics are increasingly used to characterise large-scale communication dynamics across brain networks in both cognitive and clinical domains, demonstrating their utility for identifying network topology changes beyond local power alterations (Imperatori et al., 2019).

Combining gamma power with connectivity help delineate the mechanisms of swallowing in PSD patients. A large number of studies have demonstrated that somatosensory stimulation could induce gamma activities (Hashimoto et al., 1996; Hirata et al., 2002; Muhle et al., 2021). This is in line with the role of gamma oscillation in integrating distributed neural processes (Başar et al., 2001; Luo et al., 2023). Beyond gamma power, rhythmic synchronisation of neural discharges in the gamma range is critical for the integration of sensations into a coherent state of moment-to-moment awareness (Lomas et al., 2015; Singer, 1993; Tallon-Baudry and Bertrand, 1999). Our novel findings revealed that PSD patients were characterised by hypoactivity and hypoconnectivity in gamma band during swallowing, in widely distributed regions over the frontal, central, and parietal regions. These findings highlight the role of gamma oscillation in integrating somatosensory, motor, and cognitive information to support swallowing performances. Moreover, gamma-band synchrony has been proposed as a biomarker of effective neural communication that can be modulated by non-invasive brain stimulation, making it a promising physiological indicator for future neuromodulation interventions in PSD (Rogasch and Fitzgerald, 2013; Casarotto et al., 2010). However, the gamma effects over distributed channels should be considered in the context of a sparse electrode distribution in the current study.

It is important to note that this hypoconnectivity pattern reflects the dominant network abnormality in PSD, whereas the few connections in our data showed increased gamma synchronisation. Specifically, our data demonstrated additional gamma hyperconnectivity within the frontal regions and between the frontal and occipital regions during swallowing, in the PSD patients compared to healthy controls. Given the impaired swallowing capacity, gamma hyperconnectivity in our data could possibly reflect increased but inefficient neural effort during swallowing. As gamma activity is well known to be modulated by muscular recruitment (Brown et al., 1998; Michou et al., 2012; Mima et al., 2000), gamma hyperconnectivity in PSD patients could also be attributed to excessive muscle recruitment for swallowing performances. It is important to note that gamma power (reflecting local neural activation) and gamma connectivity (reflecting inter-regional communication) index distinct physiological processes (Mateos et al., 2022; Fernandez-Ruiz et al., 2023); therefore, reductions in power can coexist with increases in connectivity without representing a contradiction. Interestingly, this pattern of gamma hyperconnectivity was associated with more severe dysphagia in our data. This finding provides direct insights into the optimisation of treatment effects on dysphagia (see discussion later). Such maladaptive increases in gamma connectivity have also been observed in other neurological conditions, where excessive synchronisation reflects inefficient cortical recruitment rather than functional enhancement, highlighting gamma network measures as potential neuromodulation targets for restoring more optimal neural communication (Uhlhaas and Singer, 2013).

It is worth noting that beta activity has often been observed during swallowing. Specifically, swallowing could come with a beta inhibition, followed by a subsequent beta rebound (Hashimoto et al., 2021b; Ogawa et al., 2022; Salmelin et al., 1995). This pattern of beta activity is also well documented in other movement tasks (Fry et al., 2016; Jensen et al., 2005; Mao et al., 2024). Our data did not reveal a significant beta abnormality during swallowing in the PSD compared to healthy individuals. Given that beta desynchronisation reflects the release of motor inhibition and is essential for coordinated movement execution, the reduced trend in beta modulation may still suggest impaired inhibitory control during swallowing in PSD. However, there was a trend for beta inhibition in the PSD patients. This null finding of beta oscillation thus should be considered in the context of a small sample size.

It is important to note that different phases of swallowing (e.g., oral, pharyngeal, and oesophageal phase) were found to be characterised by unique gamma oscillations (Hashimoto et al., 2021a; Hashimoto et al., 2021b). An electroglottograph (EGG) system could be used in the determination of different swallowing phases (Hashimoto et al., 2021b; Firmin et al., 1997). Our data of gamma hypoactivity was found mainly in the 1–2 s time frames, likely reflecting oral phases. This is particularly relevant given that impaired oral control is one of the earliest and most prevalent deficits in PSD, potentially contributing to the delayed swallow initiation and increased aspiration risk frequently reported in this population. The use of liquid boluses aligns with standardised clinical assessments for post-stroke dysphagia. Liquids pose the greatest challenge for airway protection due to their low viscosity and rapid flow, making them a sensitive probe for identifying impaired pharyngeal coordination. However, an EGG system was not available in our department, which limited our capacity to characterise gamma band abnormalities for PSD patients in each of these swallowing phases. Although laryngeal elevation markers and suprahyoid sEMG recordings were employed to establish a consistent swallow onset time for EEG epoch segmentation, these physiological signals lack the temporal resolution necessary for distinguishing distinct swallowing phases. The temporal and amplitude characteristics of sEMG activity demonstrate considerable inter-individual variability, while laryngeal elevation represents a generalised pharyngeal motor event rather than precisely marking transitions between oral, pharyngeal, and oesophageal stages. Consequently, neither signal enables reliable identification of phase boundaries, a task that generally requires specialised techniques such as electroglottography or videofluoroscopy.

Our novel findings enrich our understanding of swallowing mechanisms and provide insights to optimise treatment strategies for PSD. We identified consistent gamma band abnormalities across power and phase synchronisation. Previous clinical and meta-analytic studies have shown that repetitive transcranial magnetic stimulation (rTMS) applied over swallowing-related cortical regions can improve swallowing performance in patients with post-stroke dysphagia (Khedr et al., 2009; Wen et al., 2022) These findings support the feasibility of using neuromodulation to target pathological cortical oscillations that contribute to dysphagia (Khedr et al., 2009; Wen et al., 2022). Moreover, recent work in healthy adults has shown that prefrontal rTMS can enhance aspects of cognitive and sensorimotor function by modulating gamma-band oscillations, suggesting that gamma synchronisation is a mechanistically relevant target for stimulation. In line with this mechanistic relevance, our current findings revealed abnormal gamma activity and synchronisation patterns in PSD patients. Moreover, our data identified a unique gamma band hyperconnectivity and its association with dysphagia severity. This could be tested as a biomarker to personalise neuromodulation treatments for PSD patients, such as tDCS, tACS, rTMS, etc. (Michou et al., 2012; Khedr et al., 2009; Park et al., 2017; Yang et al., 2012). For instance, tACS at gamma frequencies over frontocentral sites could be used to normalise hypoconnectivity. Moreover, these network-level findings could inform future work exploring dual- or multi-site TMS treatment strategies (Cui et al., 2024; Liu et al., 2023; Xu et al., 2024; Wang et al., 2025). Future studies are warranted to determine whether gamma-driven targeting strategies yield superior clinical outcomes compared with conventional stimulation protocols. Although PSD–healthy comparisons have been emphasised in previous EEG swallowing studies, the clinically more meaningful contrast involves PSD patients and stroke patients without dysphagia, who share similar vascular etiologies but differ in functional outcome. The present results revealed that PSD patients exhibited not only altered gamma activity relative to healthy controls but also showed theta-band disinhibition and weakened connectivity compared to stroke patients without dysphagia. These findings suggest that abnormal swallowing-related oscillations in PSD cannot be solely attributed to stroke itself but rather may reflect dysphagia-specific impairments in neural recruitment or inhibitory control (Imaz-Higuera et al., 2025; Qin et al., 2023; Velasco-Hernandez et al., 2025). This pattern aligns with the notion that dysphagia involves disrupted integration within the cortico-bulbar swallowing network, in which excessive compensatory drive fails to restore efficient sensorimotor control. Therefore, gamma and theta oscillations may serve as neurophysiological markers that distinguish functionally relevant swallowing network degeneration from general post-stroke changes.

The strongest gamma-band abnormalities emerged when comparing PSD patients with healthy controls; however, given the limited sample size, these findings should not be interpreted as evidence that such gamma abnormalities are exclusive to PSD (Velasco-Hernandez et al., 2025; Rabiller et al., 2015; Dehghani et al., 2016). In addition, higher theta power in PSD compared to stroke controls could be linked to differences in how well people do tasks or how much effort they put in (Velasco-Hernandez et al., 2025; Lacerda et al., 2024). The PSD group has trouble in swallowing; consequently, prolonged swallow duration or cortical engagement may be indicated in theta (Li et al., 2025). However, there is no evidence to support these hypotheses (Sood et al., 2024), and future studies may wish to validate these assumptions. Importantly, the present findings provide initial evidence that theta-band abnormalities may reflect dysphagia-related mechanisms beyond general post-stroke changes.

There were some limitations in this study. The sample size may be inadequate for obtaining robust results and characterising a population of PSD with highly diverse clinical profiles. For instance, the sample size might affect power for detecting subtler effects in a trend theta effect. Based on the effect size of gamma or theta power, sample size calculations indicated a minimum 18 and 56 participants for a future study to be sufficiently power. Thus, this is considered as a pilot study and results of which need to be validated in future investigations with larger samples. Moreover, most of our PSD participants were males, which raises the possibilities of potential gender differences. It is recommended for future studies to include balanced genders. In addition, previous studies indicated the presence of lateralisation in swallowing (Teismann et al., 2009; Imaz-Higuera et al., 2025; Mosier et al., 1999; Sörös et al., 2009). This has also been debated in a recent study (Cheng et al., 2022). However, the current EEG montage used a relatively sparse electrode distribution, and group-level plots reflected averaged activity rather than hemisphere-specific effects. Therefore, hemispheric differences may not be detectable with the present setup. Ideally, an increased number of electrodes (e.g., 64) or source imaging could yield superior spatial resolution. It is worth noting that our connectivity results did not survive more robust statistical analyses, such as the cluster-based permutation across connections or network-based statistics (Maris and Oostenveld, 2007; Zalesky et al., 2010). This is largely due to the small sample size in each group and a large number of connections between sensors (171 pairs). We thus acknowledge that our connectivity profiles are more of exploratory in nature and should be validated in future large studies with network-based statistics. In conclusion, PSD patients demonstrated predominant gamma band hypoactivity and hypoconnectivity during swallowing. We also identified an excessive gamma band connectivity that was related to more severe dysphagia, possibly reflect inefficient neural effort or muscle recruitment during swallowing. Our findings help enrich our understanding of swallowing mechanisms and provide insights to optimise treatment strategies for PSD. These findings highlight gamma-band oscillatory markers as promising candidates for guiding personalised neuromodulation strategies in PSD. Nevertheless, the results should be interpreted cautiously given the sample size and validated in future larger-scale studies.

## Data Availability

The raw data supporting the conclusions of this article will be made available by the authors, without undue reservation.
